# Efficacy of magnesium sulfate as an adjuvant to local anesthetics in supraclavicular brachial plexus block: a meta-analysis of randomized trials

**DOI:** 10.1016/j.bjane.2025.844689

**Published:** 2025-10-16

**Authors:** Willyam Barros Saraiva, Isadora Eloy Candido, Roberta Ribeiro Brandão Caldas, Fabiano Timbó Barbosa

**Affiliations:** a Universidade Federal de Alagoas (UFAL), Hospital Universitário Professor Alberto Antunes, CET/SBA, Maceió, AL, Brazil; b Faculdade UNIMA, Departamento de Medicina, Maceió, AL, Brazil; c Sociedade Brasileira de Anestesiologia (SBA), Higher Degree in Anesthesiology (TSA), Rio de Janeiro, RJ, Brazil; d Universidade Federal de Alagoas (UFAL), Faculdade de Medicina, Maceió, AL, Brazil

**Keywords:** Anesthetics, local, Magnesium sulfate, Meta-analysis, Nerve block, Pain, postoperative, Systematic review

## Abstract

**Background:**

Magnesium Sulfate (MS) maintains physiological functions in the body. Studies suggest its safety in regional anesthesia, despite off-label perineural use. We conducted a systematic review and meta-analysis to evaluate MS efficacy as an adjuvant in supraclavicular brachial plexus block.

**Methods:**

The study was registered in PROSPERO (CRD42025641627) on 01/21/2025. We searched PUBMED, Embase, Cochrane, clinicaltrials.gov and gray literature for eligible studies. We included RCTs that: enrolled adult patients; involved orthopedic surgery with supraclavicular block; compared LA alone versus LA with MS; and reported primary outcomes. Primary outcomes were duration of sensory and motor block, while secondary outcomes included onset of sensory and motor block, PONV and rescue analgesia needs postoperatively. RoB2 tool and GRADE assessed bias risk and evidence certainty. Variables were examined using DerSimonian-Laird random-effects model.

**Results:**

Analysis included 10 studies and 734 patients. The intervention group showed longer sensory and motor block than controls. The Mean Difference (MD) was 180.84 minutes (95% CI [154.09, 207.59], 95% PI [71.67, 289.77], p < 0.00001, I² = 97%) and 151.26 minutes (95% CI [99.78, 202.74], 95% PI [-23.12, 325.63], p < 0.00001, I² = 99%). The magnesium group showed statistical difference in onset of sensory and motor blockade and rescue analgesia needs, with no difference in PONV. Evidence certainty was rated low to moderate. Risk of bias “high” in three studies, “some concerns” in four studies and “low” in three studies.

**Conclusion:**

Our meta-analysis supports MS as adjuvant in supraclavicular block. Further research is needed due to high heterogeneity.

**PROSPERO registration:**

CRD42025641627.

## Introduction

The supraclavicular block is a regional anesthetic technique for primary regional anesthesia during surgeries and/or postoperative pain control to the distal two-thirds of the upper extremity, or from the mid-humerus to the fingertips.^1^

Although Local Anesthetic (LA) agents provide superior analgesia compared to opioid-based regimens, their effect is time-limited and may not adequately cover the postoperative pain period.^2^ As a result, strategies to prolong the duration of single-shot nerve blocks have become a clinical priority. One such strategy is the use of perineural adjuvants ‒ pharmacologic agents co-administered with Las ‒ to extend block duration and improve analgesic quality. This approach is especially valuable in outpatient and daycare surgeries, where prolonged anesthesia may reduce the need for continuous catheter placement and lower the risk of catheter-related infections.^3^

Several agents have been studied as adjuvants to LAs, including alpha-2 adrenergic agonists and glucocorticoids. Magnesium Sulfate (MS) is an N-Methyl-D-Aspartate (NMDA) receptor antagonist that modulates pain transmission and plays an essential role in maintaining physiological homeostasis. While its perineural use remains off-label, multiple studies have suggested its safety in regional anesthesia.^4^, ^5^, ^6^

Despite promising findings, high-quality evidence remains limited regarding magnesium sulfate's efficacy and safety as an adjuvant in Peripheral Nerve Blocks (PNBs). Existing studies^6^, ^7^, ^8^ vary in block technique and outcome reporting, contributing to heterogeneity and limiting generalizability. We conducted a systematic review and meta-analysis of randomized controlled trials to evaluate magnesium sulfate as a perineural adjuvante, focusing on a widely used upper limb block^9^ – the supraclavicular block. We hypothesized that MS would prolong sensory and motor block duration, reduce block onset time, and not increase adverse effects compared to local anesthetic alone.

## Methods

The study was registered in PROSPERO (identifier CRD42025641627) on 01/21/2025 and was conducted using PUBMED, Embase, Cochrane Central Register of Controlled Trials (CENTRAL), Gray literature (opengrey.eu) and trial registries (clinicaltrials.gov) databases to identify eligible studies. During the review process, a small deviation from PROSPERO occurred: (1) The search strategy was updated to include additional descriptors, which resulted in the inclusion of more studies. Two researchers (W.B.S. and I.E.C.) independently conducted the database searches, which was completed in January 31, 2025, without imposing any limitations. Any discrepancies between the two researchers were resolved through discussions with a third author (R.R.B.C.). Supplemental Table 1 presents the detailed search strategy: ('magnesium sulfate'/exp OR 'magnesium sulfate':ti,ab) AND ('brachial plexus block'/exp OR 'brachial plexus block':ti,ab OR 'block, brachial plexus':ti,ab OR 'blocks, brachial plexus':ti,ab OR 'brachial plexus blocks':ti,ab OR 'brachial plexus anesthesia':ti,ab OR 'anesthesia, brachial plexus':ti,ab OR 'brachial plexus blockade':ti,ab OR 'blockade, brachial plexus':ti,ab OR 'blockades, brachial plexus':ti,ab OR 'brachial plexus blockades':ti,ab OR 'plexus blockade, brachial':ti,ab OR 'plexus blockades, brachial':ti,ab OR 'brachial plexus'/exp). Our study adhered to the guidelines set forth by the Preferred Reporting Items for Systematic Reviews and Meta-Analyses (PRISMA),^10^ Cochrane Handbook for Systematic Reviews of Intervention^11^ and when applicable, other generated guidelines.^12^^,^^13^

### Selection of the papers

The primary outcomes assessed were the duration of sensory and motor block. Secondary outcomes included the onset of sensory and motor block, Postoperative Nausea and Vomiting (PONV), and the need for rescue analgesia within 24 h postoperatively. Rescue analgesia was defined as the total amount of analgesic drug administered during the first 24 hours after surgery, recorded in milligrams of the specific medication used in each trial. The systematic review and meta-analysis included Randomized Controlled Trials (RCTs) that met the following criteria: 1) Enrolled adult patients; 2) Involved orthopedic surgery with a supraclavicular block; 3) Compared LA alone versus LA with MS; and 4) Reported both primary outcomes defined in this review. Studies were excluded if they 1) Included urgent or emergency surgery, or 2) Had an ASA status equal to or greater than III. The rationale for the inclusion and exclusion criteria is detailed in Supplemental Table 2.

Two researchers (W.B.S. and I.E.C.) conducted the selection after independently evaluating the studies for inclusion, based on predetermined criteria. After eliminating duplicates, the remaining results were screened according to title and abstract. The full texts of the potentially relevant studies were subsequently examined to confirm their eligibility. In cases where full texts were not readily available, efforts were made to contact the corresponding authors directly, but studies remained excluded if the necessary data could not be obtained. Abstracts that did not provide outcome information were excluded, as they could not provide data extraction. Any discrepancies between the two researchers were resolved through discussions with a third author (R.R.B.C.). Zotero Software version 7.0.15 was used to select the studies and eliminate duplicates.

### Data analysis

After these procedures, a data extraction table was constructed based on the following variables: author, publication year, country, ASA status, surgery type, MS and Local Anesthetic (LA) dosages, patient count, mean age, and volume of the mixture utilized.

The revised Cochrane Risk-of-Bias tool for randomized trials 2^14^ (RoB 2) was employed by two researchers (W.B.S. and I.E.C.) to assess the risk of bias independently. Risk of bias was classified as "low risk", "some concerns", or "high risk." Discrepancies were also resolved through discussion with a third researcher (R.R.B.C.).

The Grading of Recommendations Assessment, Development, and Evaluation (GRADE)^15^ system was employed to evaluate evidence certainty. Evidence was subsequently classified as high, moderate, low, or very low using GRADEpro software.^16^ Two researchers (W.B.S. and I.E.C.) independently performed this stratification and any disagreements were resolved through consultation with a third researcher (R.R.B.C.).

### Statistical tests

Review Manager version 5.4 (Cochrane Collaboration)^17^ and R software version 4.5.1^18^ (PWR^19^ and metafor^20^ packages) were used to conduct statistical analyses of the meta-analysis. Cochran's *Q* test and I² statistics were employed to measure heterogeneity. For outcomes exhibiting high heterogeneity (I² > 75%),^11^ leave-one-out sensitivity analysis was performed. For continuous outcomes, Mean Differences (MD) or Standardized Mean Diferences (SMD) with 95% Confidence Intervals (95% CI) were calculated, whereas Risk Ratios (RR) with 95% CI were used for binary outcomes. Furthermore, Prediction Intervals (PI) were determined to assess the treatment effect in upcoming clinical studies.^21^ Variables were examined using DerSimonian-Laird^22^ random-effects model, with statistical significance set at p < 0.05. Power analysis of sample sizes was performed to determine whether sample sizes were adequate to detect clinically significant differences. To evaluate publication bias across all outcomes, a funnel plot analysis was employed, supplemented by Egger's regression^23^ for outcomes with a minimum of ten studies.

## Results

**Figure 1:**
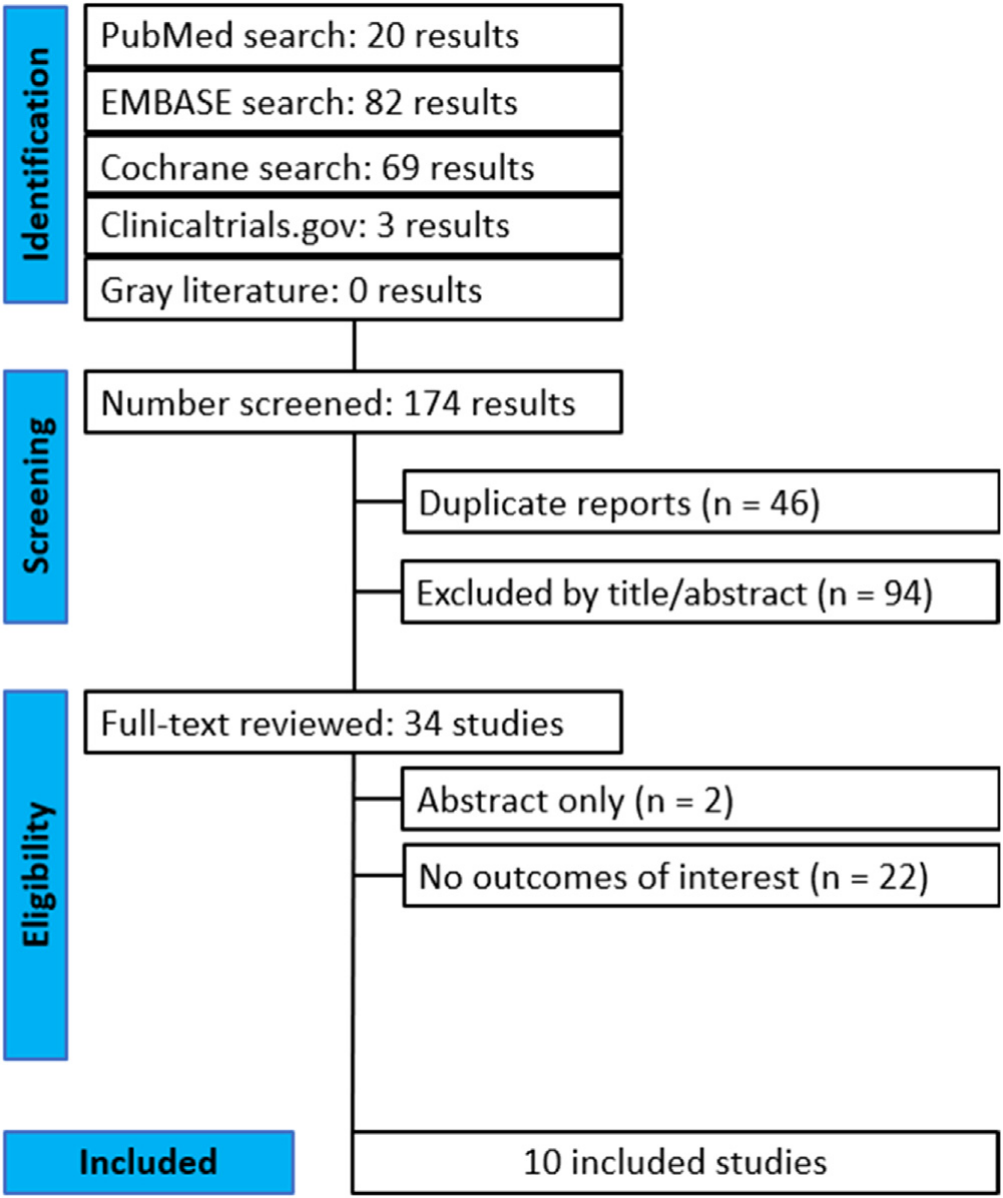
PRISMA flow diagram of study screening and selection.

Our database search strategy retrieved 174 potentially relevant records that were published up to January 2025. Of these, 46 records were excluded after initial screening for duplicate work and another 94 were excluded after reading the title and abstract. Of the 34 studies fully reviewed, 2 only provided the abstract and 22 did not contain any outcome of interest. Ten full-text randomized trials^24^, ^25^, ^26^, ^27^, ^28^, ^29^, ^30^, ^31^, ^32^, ^33^ were included in the final analysis. In the study by Verma et al.,^32^ the two intervention groups were evaluated as separate independent comparisons. Figure 1 represents the Preferred Reporting Items for Systematic reviews and Meta-Analyses (PRISMA) flow diagram and summarizes the reasons for the exclusion of records. The GRADE summary of findings for each endpoint is presented in Supplemental Table 3, with the certainty of evidence for the outcomes rated as low to moderate.

Table 1 summarizes the baseline characteristics of the included studies. Data from 734 patients, including 367 in the MS group and 367 in the control group, were available for analysis. The peripheral blocking technique was anatomical (landmark) in one trial,^24^ nerve stimulation in four trials^25^^,^^27^, ^28^, ^29^ and ultrasound in five trials.^26^^,^^30^, ^31^, ^32^, ^33^ All trials used long-acting LAs (ropivacaine or bupivacaine) and the dose of MS varied between 125 and 250 mg.

**Table 1:** Baseline characteristics of included studies.

Study	Objectives	Patients, LA vs. MS	Country	Male, %, LA vs. MS	Age, y, LA vs. MS	ASA status	Regional anesthesia technique	LA, dose	MS, dose	Choice for rescue analgesia
Aggarwal 2022	To study the effect of adding magnesium as an adjuvant to ropivacaine in supraclavicular block.	40/40	India	55/47.5	44.8 ± 6.6 vs. 45.2 ± 7.7	I-II	Landmark	Ropi 0.5%	150 mg	Diclofenac sodium
Borgohain 2023	The advantage of using magnesium sulphate as an adjuvant to bupivacaine on the postoperative analgesia as well on the onset and the duration of sensory and motor blockade in the patients undergoing upper limb surgeries and to evaluate for any possible side effects or complications.	45/45	India	NA	36.15 ± 13.44 vs. 37 ± 14.78	I-II	Nerve stimulation	Bupi 0.5%	200 mg	‒
Gupta	To compare the effectiveness of addition of MgSO_4_ (150 mg) and fentanyl (50 micrograms) to 0.375% bupivacaine with placebo in supraclavicular brachial plexus block.	25/25	India	64/64	31.17 ± 11.91 vs. 36.00 ± 13.01	I-II	Ultrasound-Guided	Bupi 0.375	150 mg	Diclofenac sodium
Jalili 2024	To compare the effectiveness of adding Magnesium Sulfate (MS) and Low-Dose Dexamethasone (LDD) to ropivacaine in SCBPBs for elective upper extremity surgery.	15/15	Iran	73.3/80	42.73 ± 12.41 vs. 46.73 ± 13.30	I-II	Nerve stimulation	Ropi 0.5%	200 mg	Opioid
Kaur 2019	To evaluate the effect of MgSO_4_ compared to ketamine when added to 0.5% ropivacaine for supraclavicular brachial plexus block, in terms of the duration of postoperative analgesia in adult patients undergoing upper limb surgery.	34/34	India	80/85.7	38.80 ± 14.37 vs. 45.22 ± 11.71	I-II	Nerve stimulation	Ropi 0.5%	250 mg	Diclofenac sodium
Mukherjee 2014	To test the hypothesis that magnesium when added as an adjuvant to ropivacaine in supraclavicular brachial plexus block may enhance the duration of sensory and motor block, duration of analgesia, and quality of block.	50/50	India	52/64	40.5 ± 13.2 vs. 44.9 ± 11.4	I-II	Nerve stimulation	Ropi 0.5%	150 mg	Diclofenac sodium
Patel 2023	To evaluate the efficacy of magnesium when added to ropivacaine in supraclavicular brachial plexus block.	30/30	India	56.7/43.3	37.13 ± 10.41 vs. 35.53 ± 9.98	I-II	Ultrasound-Guided	Ropi 0.5%	150 mg	Diclofenac sodium
Shukla 2021	To compare the efficacy of dexmedetomidine and MgSO_4_ as an adjuvant to ropivacaine in ultrasound-guided supraclavicular brachial plexus block for upper limb surgeries in terms of onset, duration of sensory and motor blocks, and duration of analgesia.	19/19	India	73.3/68.4	35.90 ± 12.19 vs. 40.85 ± 11.20	I-II	Ultrasound-Guided	Ropi 0.5%	250 mg	Diclofenac sodium
Verma 2017	To evaluate the efficacy of MgSO_4_ in two doses (125 mg and 250 mg) as an adjuvant to bupivacaine in USG-guided supraclavicular brachial plexus block.	30/30 (a) + 30 (b)	India	60/66.6	36.93 ± 12.12 vs. 38.37 ± 13.79	I-II	Ultrasound-Guided	Bupi 0.5%	125 and 250 mg	Diclofenac sodium
Youssef 2024	To evaluate the effectiveness of MgSO_4_ and dexmedetomidine as adjuvants to ropivacaine in supraclavicular brachial plexus block.	49/49	Dubai	61.2/63.2	45.00 ± 10.50 vs. 44.00 ± 11.00	I-II	Ultrasound-Guided	Ropi 0.5%	250 mg	Parecoxib and paracetamol

ASA, American Society of Anesthesiologists; Bupi, Bupivacaine; MS, MS, LA, Local Anesthetics; NA, Not Available; Ropi, Ropivacaine.

Supplemental Figure 1 shows the risk of bias assessment for each primary outcome. The overall risk was classified as “high” in three studies,^24^^,^^25^^,^^33^ “some concerns” in four studies^27^^,^^28^^,^^30^^,^^31^ and “low” in the remaining three studies.^26^^,^^29^^,^^32^ Most studies were either double- or triple-blind,^26^, ^27^, ^28^, ^29^^,^^31^^,^^32^ with the exception of one single-blind study.^30^ However, three studies^24^^,^^25^^,^^33^ did not provide a detailed account of the blinding methodology employed, and three other studies^27^^,^^31^^,^^33^ reported participant attrition following randomization. The power analysis of sample sizes (power target: 0.8; significance 0.05) are available in Supplemental Table 4.

### Block duration

**Figure 2:**
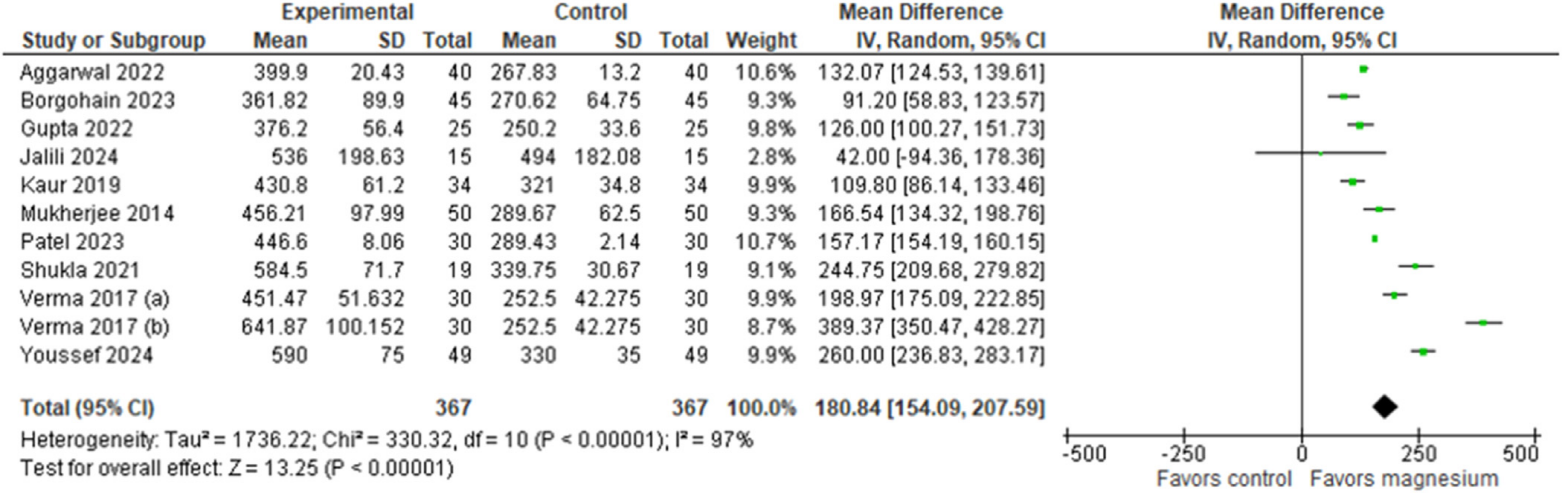
Duration of sensory block (minutes).

**Figure 3:**
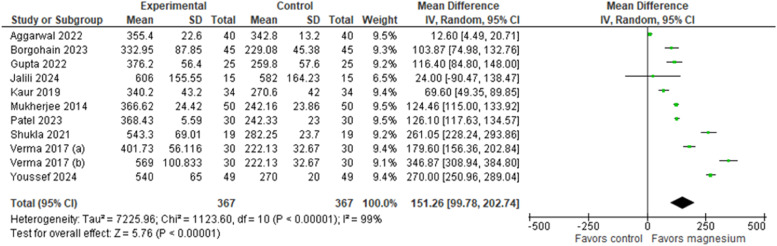
Duration of motor block (minutes).

The duration of sensory and motor blocks was reported in all ten studies,^24^, ^25^, ^26^, ^27^, ^28^, ^29^, ^30^, ^31^, ^32^, ^33^ totaling 734 patients. The results indicated that the group that used LA with the addition of MS presented a significantly longer sensory blockade time than the control group (Fig. 2), which used LA alone. The MD was 180.84 minutes (95% CI: [154.09, 207.59], 95% PI: [71.67, 289.77], p < 0.00001, Egger’s regression P = 0.4298, I² = 97%, GRADE moderate). Similarly, the use of MS as an adjunct to LA significantly improved motor block compared with the control group (Fig. 3). The pooled estimate of MD was 151.26 minutes (95% CI: [99.78, 202.74], 95% PI: [-23.12, 325.63], p < 0.00001, Egger’s regression P = 0.7826, I² = 99%, GRADE low).

A subgroup analysis was performed to estimate the relationship between dose variations and block duration, in addition to increasing the robustness of the results presented (Supplemental Fig. 2 and 3). The results showed a statistically significant difference in all subgroups of both analyses, although it is possible to observe an important variation in the MD comparing the different doses.

### Secondary outcomes

**Figure 4:**
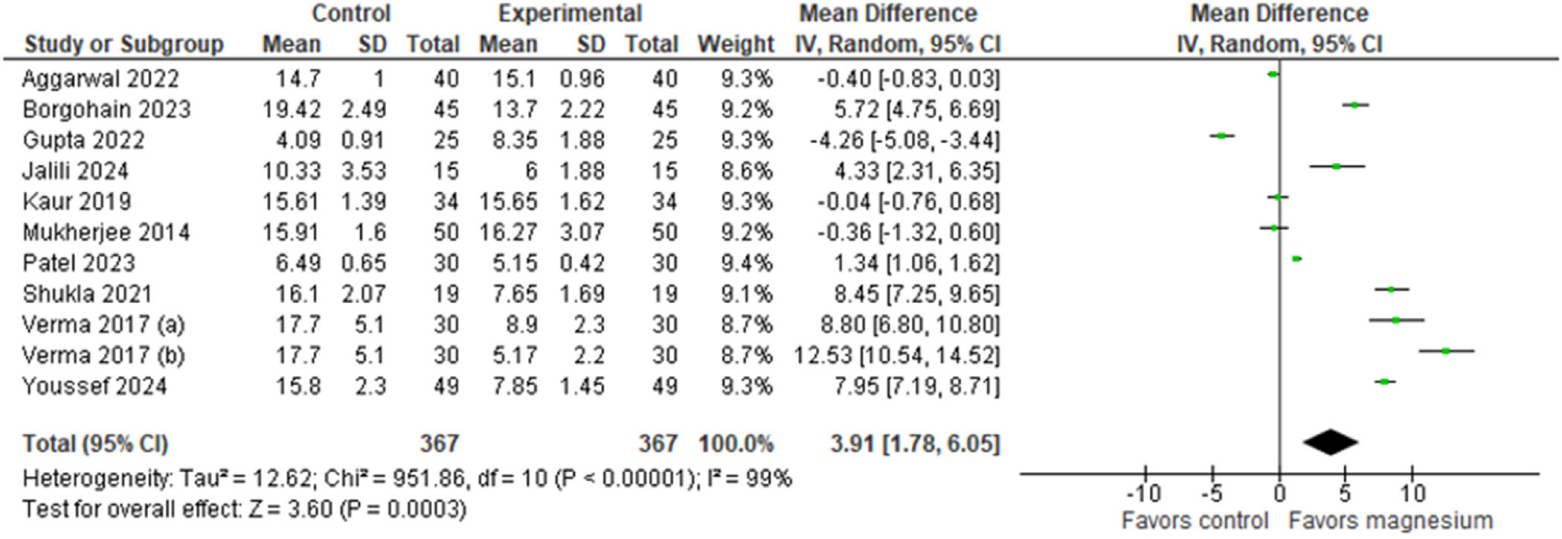
Onset of sensory block (minutes).

**Figure 5:**
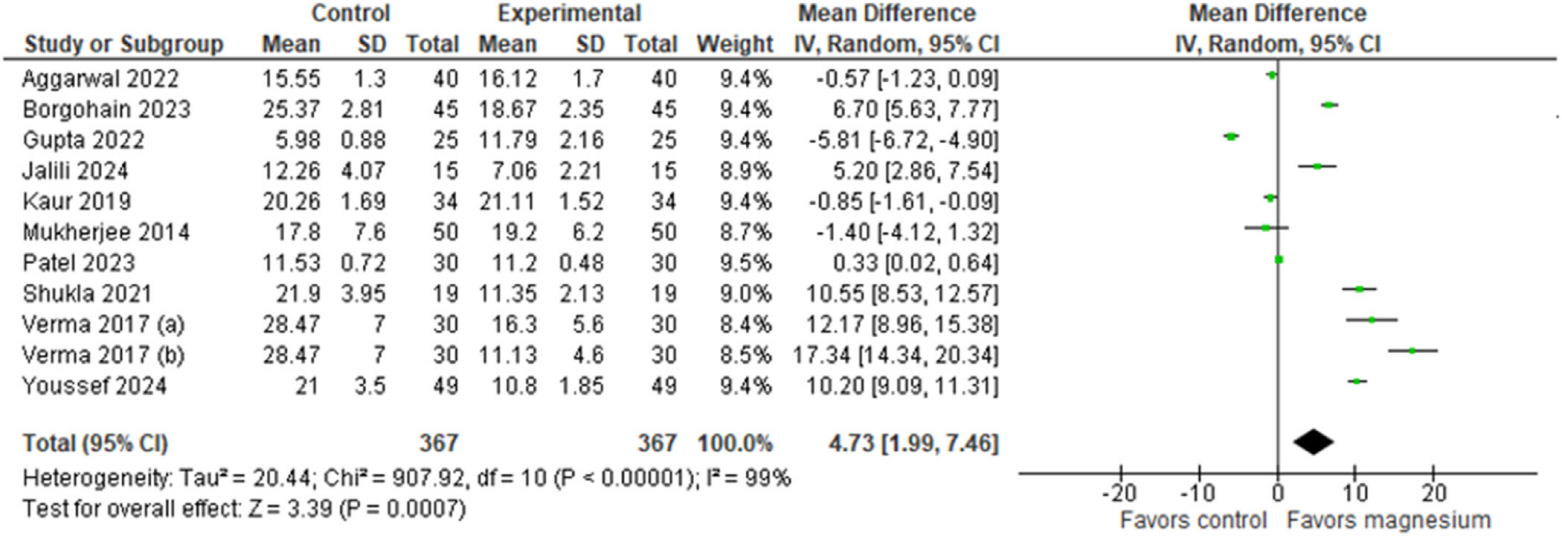
Onset of motor block (minutes).

Similarly, the onset of sensory and motor blockade was reported in all included studies^24^, ^25^, ^26^, ^27^, ^28^, ^29^, ^30^, ^31^, ^32^, ^33^ (Figure 4, Figure 5, respectively). Comparison of the time to onset of sensory blockade between the intervention group (MS + LA) and the control group (LA only) demonstrated a MD of 3.91 minutes (95% CI: 1.78, 6.05; 95% PI: -3.39, 11.22; Egger’s regression P = 0.0038 I² = 99%, GRADE low), significantly favoring the intervention group. Similarly, comparing the onset of motor blockade, the analysis showed a MD of 4.73 minutes (95% CI: [1.99, 7.46]; 95% PI: [-4.53, 13.98]; Egger’s regression P = 0.0029; I² = 99%, GRADE low), also favoring the intervention group. In the subgroup analysis (Supplemental Figs. 4 and 5), it is possible to observe a statistically significant difference favoring the MS group at doses of 200 and 250 mg in both figures.

Cumulative 24 h postoperative analgesic consumption was reported in four trials.^27^^,^^29^^,^^31^^,^^33^ In these studies, rescue analgesia was quantified as the total amount of analgesic drug administered within the first 24 hours after surgery, expressed in milligrams of the specific drug used in each trial (opioids for Jalili et al.;^27^ diclofenac sodium for Mukherjee et al.^29^ and Shukla et al.;^31^ parecoxib and paracetamol for Youssef et al.).^33^ For meta-analysis, these continuous measures were standardized and pooled, resulting in a SMD of 0.96 (95% CI: [0.36, 1.57], 95% PI: [-2.19, 0.27], p < 0.00001, I² = 80%, GRADE moderate), indicating a statistically significant reduction in postoperative analgesic consumption in the intervention group compared with control.

Another outcome analyzed was the presence of nausea or vomiting during the postoperative period (Supplemental Fig. 7). There was no statistically significant difference between the groups, with a risk ratio of 1.39 (95% CI: [0.57, 3.38], 95% PI: [0.20, 9.76], p < 0.00001, I² 0%, GRADE moderate). No significant hemodynamic changes were observed in the analyzed studies.

### Heterogeneity

The observed asymmetry in the funnel plots (Supplemental Fig. 8 and 9), especially in the primary outcomes, indicates the potential presence of publication bias or other biases, such as methodological heterogeneity among the studies. Egger's test (Supplemental Fig. 10) yielded a p-value exceeding 0.05 for the primary outcomes, indicating minimal evidence of publication bias. Conversely, the outcomes related to the onset of sensory and motor blockade demonstrated a p-value less than 0.05, suggesting an elevated risk of publication bias.

A leave-one-out sensitivity analysis (Supplemental Fig. 11) was performed under a random-effects model for all outcomes with high heterogeneity, which was applied to all endpoints, except PONV. After analyzing each outcome, no single study was responsible for the observed high heterogeneity in most outcomes. However, for the outcome “total analgesic in 24 h”, the study by Mukherjee et al.^29^ substantially contributed to the heterogeneity.

## Discussion

The main findings of our meta-analysis are as follows: 1) An extended duration of sensory and motor blockades; 2) A reduction in latency time for both motor and sensory blockades; 3) A decrease in analgesic consumption within the first 24 hours postoperatively, which in the included trials was measured as the total amount of the specific analgesic drug administered (opioid, diclofenac sodium, parecoxib, or paracetamol); and 4) No increase in PONV during the analyzed period.

The increased use of PNBs in surgeries has been a remarkable trend in recent years, with several studies highlighting the benefits of this approach compared to other anesthesia techniques.^34^ This technique may offer numerous advantages, including effective analgesia, reduced opioid consumption, lower complication rates, and a more favorable recovery profile.^35^^,^^36^

The mechanism of action by which MS potentiates the analgesic effect of LAs remains not entirely clear,^37^ although a meta-analysis has proven that the combination of MS and LAs in-nerve blocks could result in longer postoperative analgesia.^8^ Magnesium acts as an antagonist of N-Methyl-D-Aspartate (NMDA) receptors and has been shown to raise the excitation threshold in peripheral nerves, particularly in myelinated Ab fibers, compared to unmyelinated C fibers. When administered perineurally, its mechanism of action may involve the influence of its positive divalent charge on the neuronal membrane, or its function as a physiological calcium antagonist.^2^ Although its perineural use is still off-label and there are concerns about neurotoxicity, the available literature does not provide conclusive evidence of significant neurotoxic effects when MS is used as an adjunct in perineural applications.

The results showed that MS significantly increased the duration of sensory and motor blocks. These findings align with those of other studies indicating that MS, acting as an NMDA receptor antagonist, prolongs analgesic and anesthetic effects.^38^^,^^39^ A study by Ramegowda et al.^40^ reported similar results, with a significant increase in the sensory block duration in patients who received MS as an adjuvant. This effect can be attributed to the ability of MS to modulate nociceptive stimulus transmission and prolong neuronal block. The confidence interval exhibited considerable variance, alongside notably high reported effect sizes. The observed findings are likely attributable to the considerable heterogeneity across studies or perhaps to the variability in the temporal dynamics of MS when used as an adjunct. The subgroup analysis suggests a dose-dependent relationship between magnesium sulfate and anesthetic efficacy, particularly in sensory block duration. The 250 mg dose showed the greatest mean effect in both outcomes and was statistically superior to the 150 mg and 200 mg doses in prolonging sensory block (p = 0.0003). However, this trend did not reach statistical significance for motor block duration (p = 0.10), indicating the dose-response relationship varies across clinical parameters. The high heterogeneity observed may reflect methodological differences between studies ‒ such as variations in block technique, anesthetic formulations, timing of assessment, or outcome definitions. While data suggest increased efficacy with higher doses, discrepancies across subgroups may be partially influenced by methodological limitations of the included studies.

Despite its benefits, motor block prolongation and phrenic nerve involvement remain important considerations with supraclavicular block. Phrenic nerve block following this technique occurs in 0–67% of cases in clinical trials.^41^, ^42^, ^43^, ^44^, ^45^, ^46^ This risk relates to the anatomical proximity of the brachial plexus to the phrenic nerve at the supraclavicular fossa and may be influenced by local anesthetic volume and technique. While healthy individuals tolerate transient hemidiaphragmatic paresis without significant symptoms, patients with respiratory disease, obesity, or reduced cardiopulmonary function may experience respiratory compromise.^47^^,^^48^ In such cases, alternative approaches or modifications ‒ such as reducing local anesthetic volume ‒ may help minimize phrenic nerve involvement while maintaining effective analgesia.

Regarding onset time, the data indicate a significant reduction in the onset time of sensory and motor blocks with MS use. These findings are consistent with those in the literature, suggesting that MS enhances the effect of LAs, accelerating nerve blockade. Li et al.^6^ also reported a significant reduction in block onset time in patients undergoing peripheral blocks with MS. This effect can be explained by the ability of the adjuvant to alter neuronal excitability and facilitate LA diffusion. In subgroup analysis, only the 150 mg dosage did not show a statistically significant difference favoring the intervention group. Subgroup analyses showed a consistent trend of increased clinical efficacy with higher doses of magnesium sulfate in accelerating motor and sensory block onset. Doses of 200 mg and 250 mg had statistically significant effects in both outcomes, whereas 150 mg did not. The subgroup difference tests were significant for both parameters (p < 0.001), suggesting a dose-dependent response. However, high heterogeneities were also observed in both analyses.

The analysis of the need for rescue analgesia within the first 24 h postoperatively revealed a significant difference favoring the group that received MS. This reduction in rescue analgesia reflects the prolonged analgesic effect of adjuvants. Previous studies, such as those by Wu et al.,^49^ also observed lower opioid consumption postoperatively in patients receiving MS, reinforcing its role in multimodal analgesia. Regarding the need for long-term analgesia, the studies analyzed did not evaluate this topic.

The results showed no significant difference in the incidence of PONV between the groups. This finding suggests that MS does not directly affect the outcome. Although some studies have proposed that MS may reduce PONV incidence owing to its role in reducing opioid consumption,^50^ our results do not support this hypothesis.

Our meta-analysis also revealed that magnesium as an adjuvant does not appear to be associated with significant unstable changes in hemodynamic parameters such as blood pressure and heart rate. Similar outcomes have been reported in recent studies conducted for infraclavicular brachial plexus nerve block,^51^ axillary brachial plexus block^52^ and laparotomy surgery.^53^

The potential neurotoxicity of MS as an adjuvant in PNBs is the subject of ongoing investigation. Cheng et al.^54^ suggest that glutamate can increase the intracellular magnesium concentration, which may cause neurotoxicity. Animal studies have shown that the intrathecal administration of magnesium can cause nerve damage.^55^^,^^56^ In a human study, Peng et al.^45^ observed no such adverse effects after the administration of 400 mg of MS in the quadratus lumborum block. Moreover, a systematic review of neuraxial MS, which exhibits certain similarities with perineural applications, reported no significant neurological complications, although the risk has not been fully delineated.^57^ In the studies included in our analysis, no adverse effects related to neurotoxicity were reported.

The methodological quality of the included studies varied considerably. Although some trials were rated as having a low overall risk of bias, several exhibited methodological concerns or a high risk of bias. For instance, studies by Aggarwal et al.,^24^ Borgohain et al.,^25^ and Youssef et al.^33^ were classified as high risk due to the lack of blinding procedures. Jalili et al.,^27^ Kaur et al.,^28^ and Shukla et al.^31^ showed risk of bias related to post-randomization attrition, while Patel et al.^30^ presented “some concerns” due to the implementation of only single blinding. When assessed using the GRADE approach, the certainty of evidence ranged from low to moderate across most outcomes. Downgrading was primarily due to risk of bias, imprecision (evidenced by wide confidence and prediction intervals), and inconsistency among study results. Although statistically significant MDs were observed, the heterogeneity and broad prediction intervals indicate a high degree of variability in the expected effects, suggesting that individual future studies could find no effect or even effects in the opposite direction. The presence of wide prediction intervals in several outcomes highlights the need for future high-quality, well-powered randomized trials with rigorous methodology and standardized outcome reporting to strengthen the evidence on the use of magnesium sulfate as an adjuvant in regional anesthesia. Such efforts are essential to confirm the observed dose-response relationship and to reduce the risk of overestimating the effects in future evidence syntheses.

The use of MS as an adjunct in supraclavicular brachial plexus block appears to be clinically applicable, particularly for prolonging the duration of analgesia and reducing postoperative pain and analgesic consumption. PNBs associated with MS represent a viable option for patients who are at elevated risk of experiencing respiratory depression, opioid addiction, or opioid-induced nausea and vomiting,^58^ mainly in the context of upper limb surgeries, where a high incidence of postoperative pain is observed. When compared with other adjuvants, such as alpha-2 adrenergic agonists – concerns about hypotension and bradycardia –^59^ our results did not observe such adverse effects with MS, although its use is still off-label.

Based on subgroup analyses, the 250 mg dose of MS showed statistically significant superiority in prolonging sensory and motor block duration and reducing onset time versus lower doses. This dose was not associated with clinically relevant adverse effects in the studies. These findings support recommending 250 mg as the optimal dose for enhancing supraclavicular brachial plexus blocks. The dose-dependent gradient and favorable safety profile reinforces this dosage's clinical viability in routine anesthetic practice, considering patient-specific factors.

### Limitations

First, the overall methodological quality of the included trials was suboptimal, with most studies presenting some concerns or high risk of bias, particularly in randomization procedures and selective outcome reporting, as assessed by the RoB2 tool. Second, the certainty of evidence was rated as low to moderate using the GRADE approach, mainly due to serious risk of bias, inconsistency, and imprecision.

Second, high heterogeneity was observed in several outcomes, which suggests potential methodological differences among the studies. The observed differences can be attributed to several factors, including the type of surgery (such as forearm and upper limb procedures), the dosage of MS, the characteristics of local anesthetics (including types and volumes), the techniques employed for nerve blocks (such as anatomical, nerve stimulator, and ultrasound methods), and the scales utilized for outcome measurement. Despite these findings, no single study was responsible for the observed high heterogeneity in most of the outcomes (except Mukherjee et al.^37^ in “total analgesic in 24 h”). In addition, some results report very wide confidence intervals, indicating variability in effect size estimates and possible inconsistencies.

Third, the individual trials had small sample sizes, ranging from 15 to 60 patients per group, which increased the risk of type I error and publication bias. However, after conduct a power analysis, we observed these sample sizes are adequate to detect clinically significant differences. Furthermore, our results are based on a limited number of studies (10).

Fourth, most of the studies were conducted in India, limiting generalizability. The findings of our research may have been influenced by ethnic or geographical commonalities.

### Strengths

Our study possesses several notable strengths. We conducted a comprehensive literature search across major databases and trial registries, including gray literature sources, to minimize publication bias. The inclusion criteria were strictly limited to RCTs, which enhances the methodological rigor and validity of the findings. In addition, we performed subgroup analyses to explore potential sources of heterogeneity and improve the robustness of our conclusions. To further assess the risk of publication bias, we conducted both funnel plot inspection and Egger’s regression asymmetry test, providing greater transparency in our synthesis. Methodologically, we applied the RoB2 tool to evaluate study quality in a structured, domain-based manner, and conducted a leave-one-out sensitivity analysis to determine the influence of individual studies on the pooled estimates. Finally, we used the GRADE approach to construct a summary of findings table and assess the certainty of evidence for each outcome, thereby enhancing the interpretability and clinical relevance of our results. These combined strategies support the internal validity of this meta-analysis.

## Conclusion

Our meta-analysis supports the use of MS as adjuvant in supraclavicular block, with positive effects on several clinical outcomes, including prolonged block duration, faster onset time, and reduced need for rescue analgesia without important hemodynamic changes or increased PONV. The results endorse the suggestion of using a 250 mg dose as the most effective for improving supraclavicular brachial plexus blocks. However, for now, generalization of the results should be done with caution due to the high heterogeneity presented in our results.

Further studies are needed to explore variables, such as other surgical settings, different nerve block techniques, and their impact on outcomes. Standardized protocols will contribute to a broader clinical applicability and a better understanding of the safety and effects of perineural use of MS.

## Data availability statement

The datasets generated and/or analyzed during the current study are available from the corresponding author upon reasonable request.

## Declaration of competing interest

The authors declare no conflicts of interest.

## Appendix Supplementary materials

Image, application 1

## Footnotes

Supplementary material associated with this article can be found in the online version at doi:10.1016/j.bjane.2025.844689.
