# Supplementary material for: Efficacy of magnesium sulfate as an adjuvant to local anesthetics in supraclavicular brachial plexus block: a meta-analysis of randomized trials
Source: Braz J Anesthesiol. 2025 Oct 16;76(1):844689. doi: 10.1016/j.bjane.2025.844689 (PMC12639445; doi:10.1016/j.bjane.2025.844689)

**BJAN-D-25-00087_Supplementary Material**

**Contents**

**Supplementary Table 1** Detailed search strategy and results for each database.

**Supplementary Table 2** Inclusion and exclusion criteria.

**Supplementary Table 3** GRADE summary of findings table.

**Supplementary Figure 1** Risk of Bias Assessment.

**Supplementary Table 4** Power analysis of sample sizes.

**Supplementary Figure 2** Forest plot of the duration of sensor block subgrouped according to dose of MS.

**Supplementary Figure 3** Forest plot of the duration of motor block subgrouped according to dose of MS.

**Supplementary Figure 4** Forest plot of the onset of sensor block subgrouped according to dose of MS.

**Supplementary Figure 5** Forest plot of the onset of motor block subgrouped according to dose of MS.

**Supplementary Figure 6** Forest plot for the outcome of total analgesic in 24 h.

**Supplementary Figure 7** Forest plot for the outcome of postoperative nausea and vomiting.

**Supplementary Figure 8** Funnel plots for the primary outcomes: duration of sensory block (Fig. S8a) and duration of motor block (Fig. S8b).

**Supplementary Figure 9** Funnel plots for the secondary outcomes: onset of sensory block (Fig. S9a), onset of motor block (Fig. S9b), total analgesic in 24 h (Fig. S9c), and postoperative nausea and vomiting (Fig. S9d).

**Supplementary Figure 10** Egger’s Test.

**Supplementary Figure 11.** Leave-one-out sensitivity analysis.

**Supplementary Table 1** Detailed search strategy and results for each database. The same search strategy was applied to all databases. The final search was performed in January 2025.

| **Search Strategy:** | **('magnesium sulfate'/exp OR 'magnesium sulfate':ti,ab) AND ('brachial plexus block'/exp OR 'brachial plexus block':ti,ab OR 'block, brachial plexus':ti,ab OR 'blocks, brachial plexus':ti,ab OR 'brachial plexus blocks':ti,ab OR 'brachial plexus anesthesia':ti,ab OR 'anesthesia, brachial plexus':ti,ab OR 'brachial plexus blockade':ti,ab OR 'blockade, brachial plexus':ti,ab OR 'blockades, brachial plexus':ti,ab OR 'brachial plexus blockades':ti,ab OR 'plexus blockade, brachial':ti,ab OR 'plexus blockades, brachial':ti,ab OR 'brachial plexus'/exp)** |
| --- | --- |
| **Database** | **Number of Results** |
| **PUBMED** | 20 |
| **Embase** | 82 |
| **Cochrane** | 69 |
| **Clinicaltrials.gov** | 3 |
| **Gray literature** | 0 |

**Supplementary Table 2** Inclusion and exclusion criteria.

INCLUSION CRITERIA

| **Criteria** | **Justificative** |
| --- | --- |
| adult patients | To reduce heterogeneity between studies |
| orthopedic surgery | surgeries with similar pain stimulus |
| compared LA alone versus LA with magnesium sulfate | self-explanatory |
| reported primary outcomes | self-explanatory |

EXCLUSION CRITERIA

| **Criteria** | **Justificative** |
| --- | --- |
| urgent or emergency surgery | Risk of confounding bias in the data analyzed |
| ASA status equal to or greater than III | Patient with a higher chance of adverse events in the postoperative period (risk of confoundind bias) |

**Supplementary Table 3** Grading of Recommendations Assessment, Development, and Evaluation (GRADE) table of certainty of evidence for the outcomes.

**
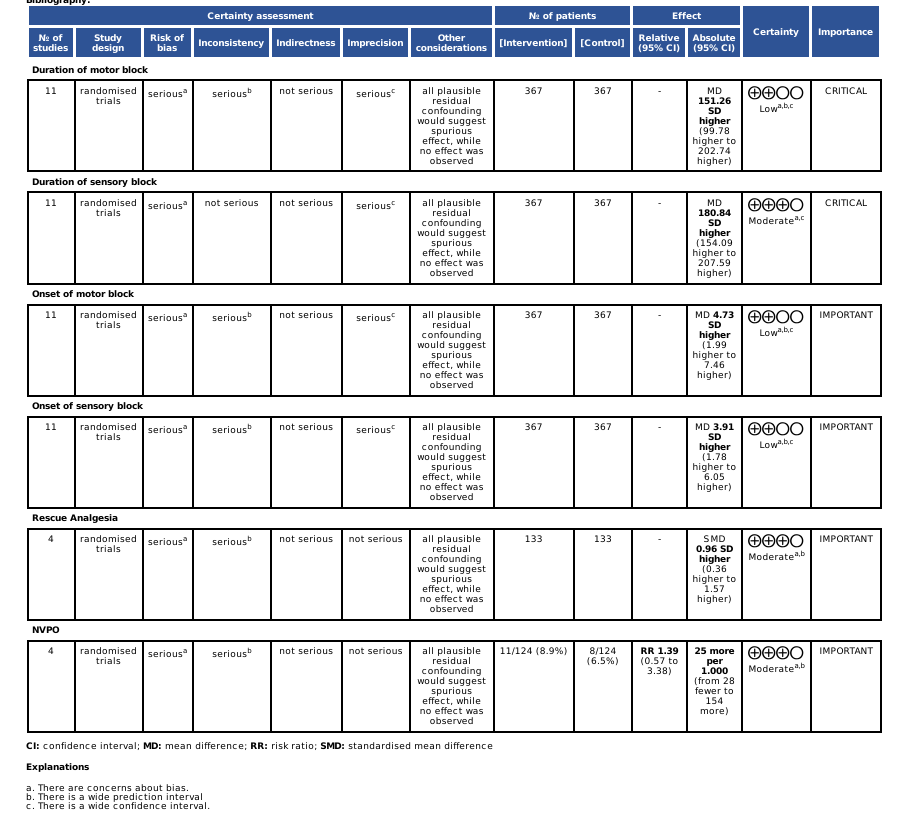
**

**Supplementary Figure 1** Risk of Bias Assessment.

**
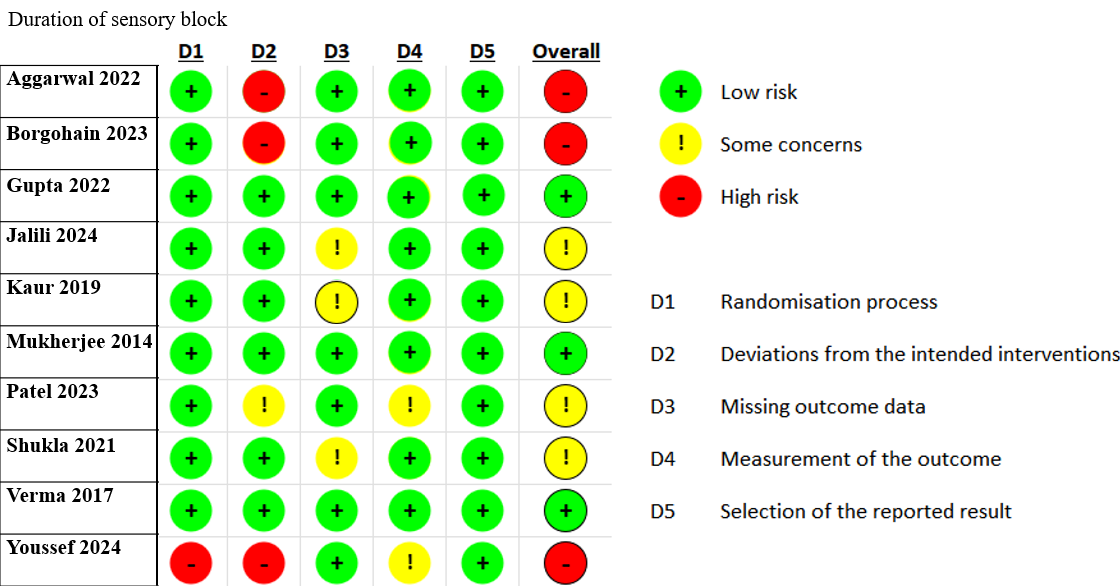
**

**
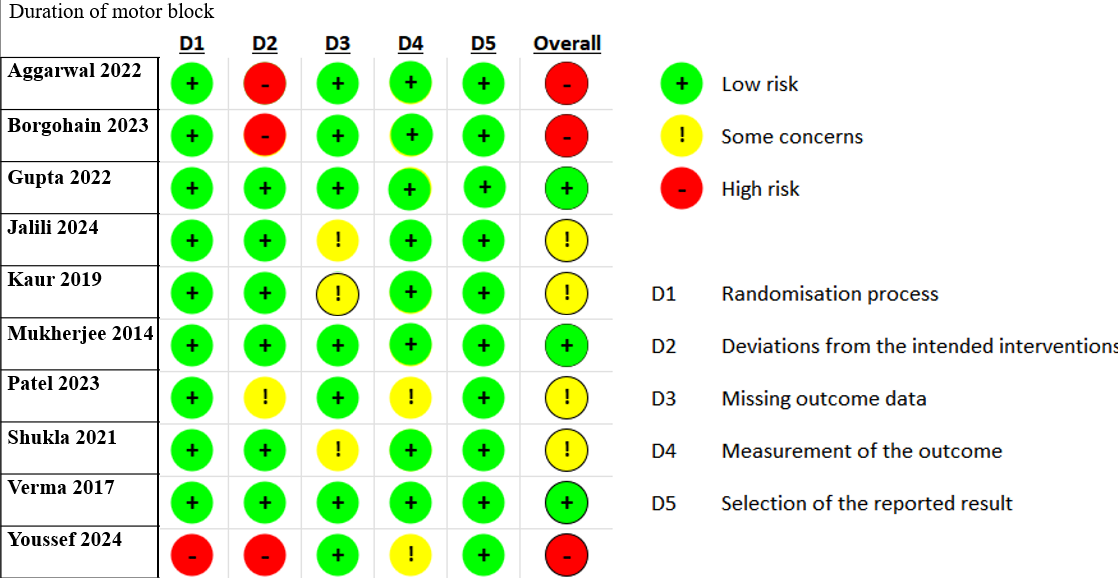
**

**Supplementary Table 4** Power analysis of sample sizes.

| **Outcome** | **Effect Size (MD)** | **Sample Size Needed for 80% Power** |
| --- | --- | --- |
| Onset of motor block | 4.73 | ~15 per group (~30 total) |
| Onset of sensory block | 3.91 | ~13 per group (~27 total) |
| Duration of motor block | 151.26 | ~5 per group (~10 total) |
| Duration of sensory block | 180.84 | ~1 per group (~2 total) |

**Supplementary Figure 2** Forest plot of the duration of sensor block subgrouped according to dose of MS.


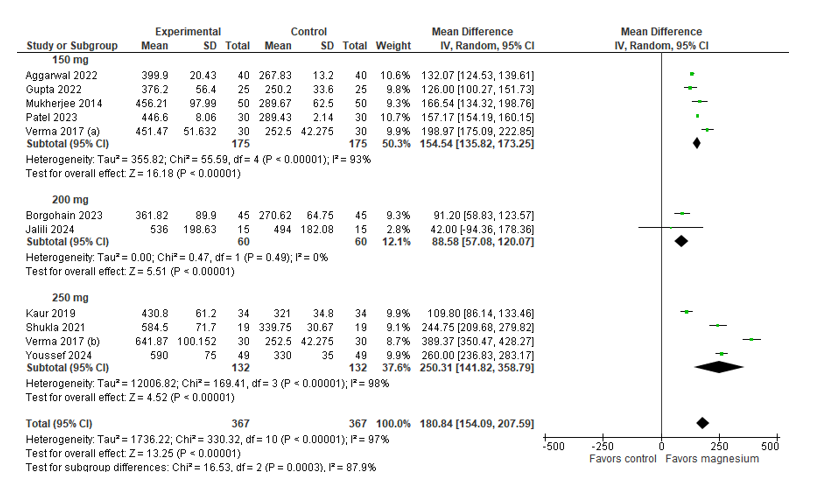


**Supplementary Figure 3** Forest plot of the duration of motor block subgrouped according to dose of MS.


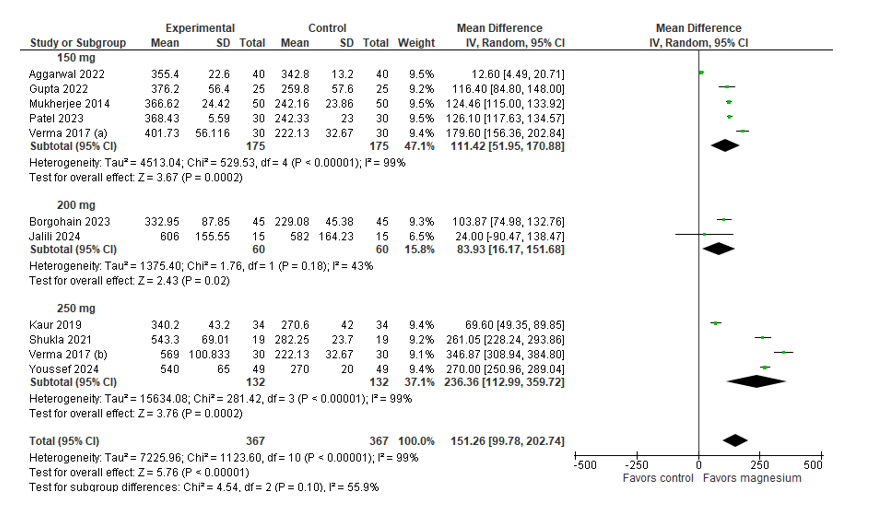


**Supplementary Figure 4** Forest plot of the onset of sensor block subgrouped according to dose of MS.


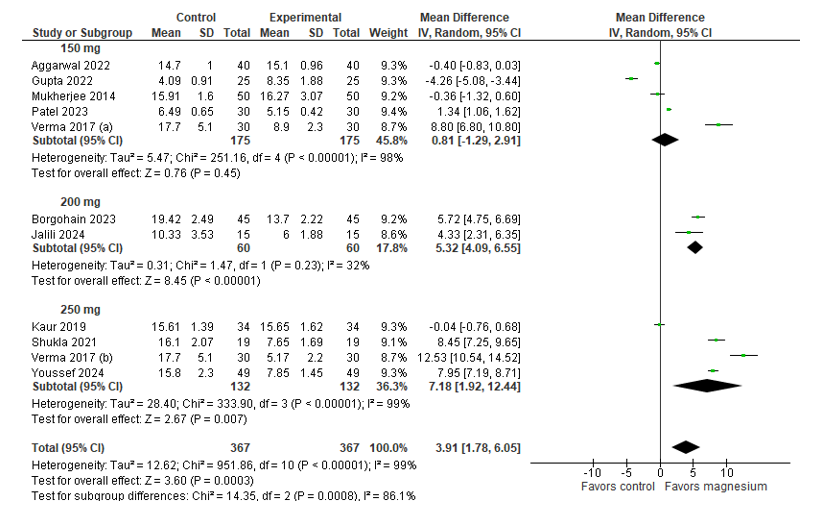


**Supplementary Figure 5** Forest plot of the onset of motor block subgrouped according to dose of MS.


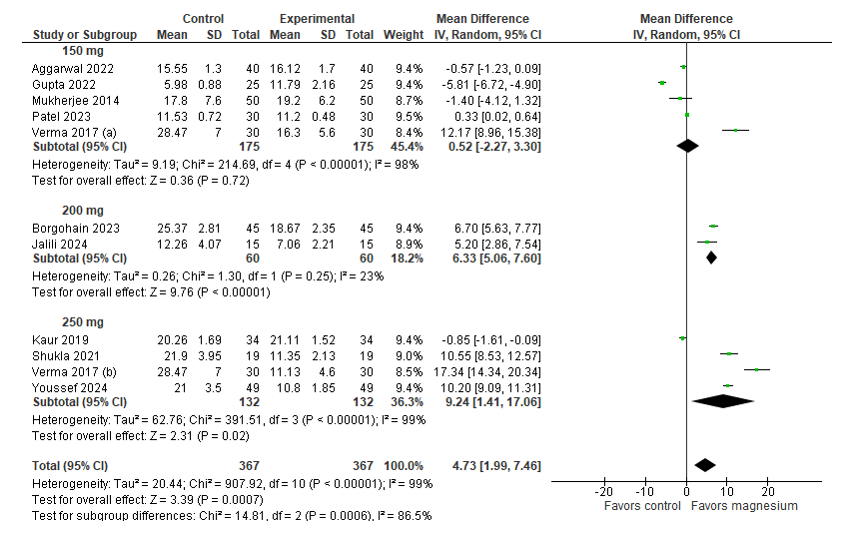


**Supplementary Figure** **6** Forest plot for the outcome of total analgesic in 24 h.


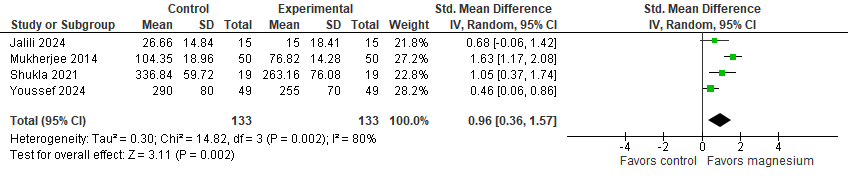


**Supplementary Figure 7** Forest plot for the outcome of postoperative nausea and vomiting.

**
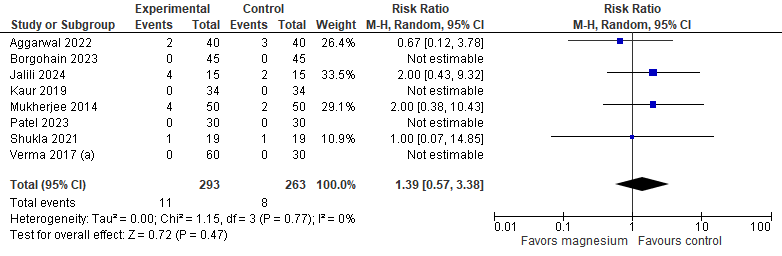
**

**Supplementary Figure 8** Funnel plots for the primary outcomes: duration of sensory block (Fig. S8a) and duration of motor block (Fig. S8b).


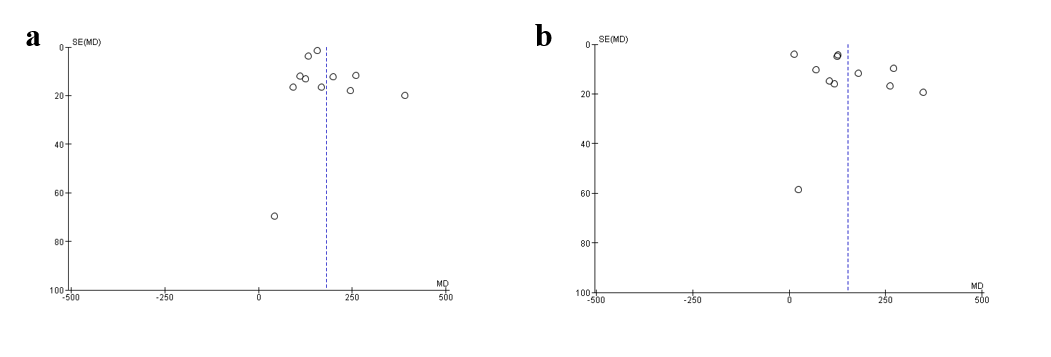
 **Supplementary Figure 9** Funnel plots for the secondary outcomes: onset of sensory block (Fig. S9a), onset of motor block (Fig. S9b), total analgesic in 24 h (Fig. S9c), and postoperative nausea and vomiting (Fig. S9d).


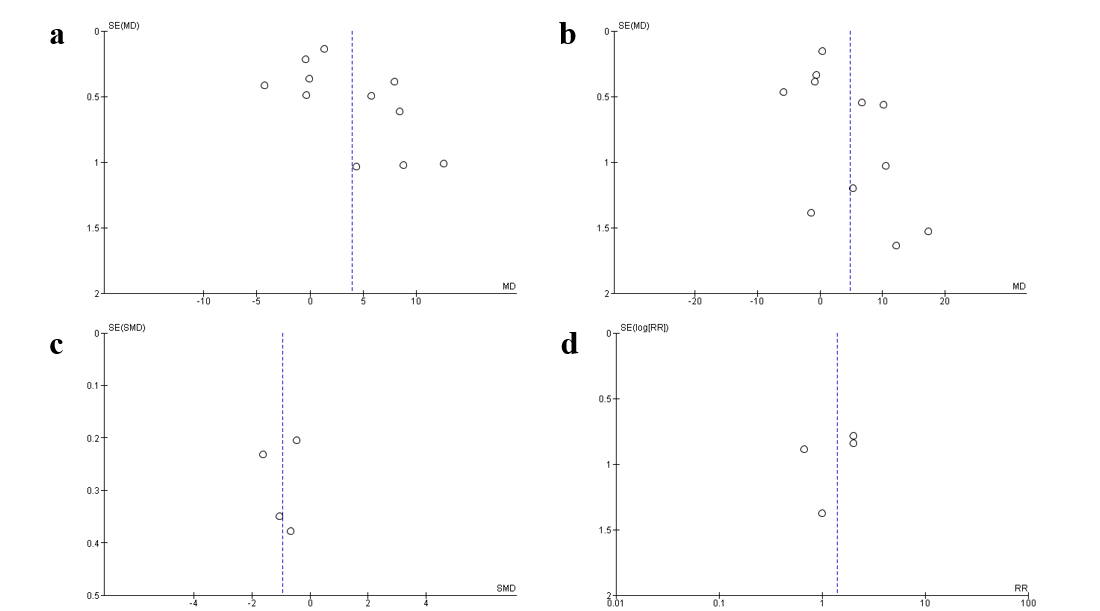


**Supplementary Figure 10** Egger’s Test.

Duration of sensory block


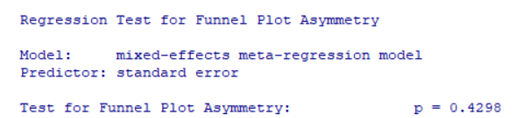


Duration of motor block


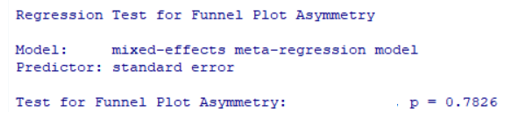


Onset of sensory block


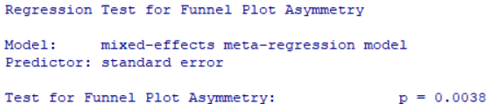


Onset of motor block


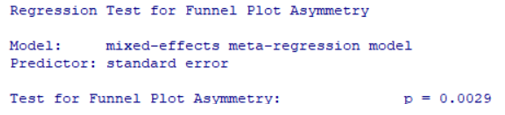


**Supplementary Figure 11** Leave-one-out sensitivity analysis.

Duration of sensory block


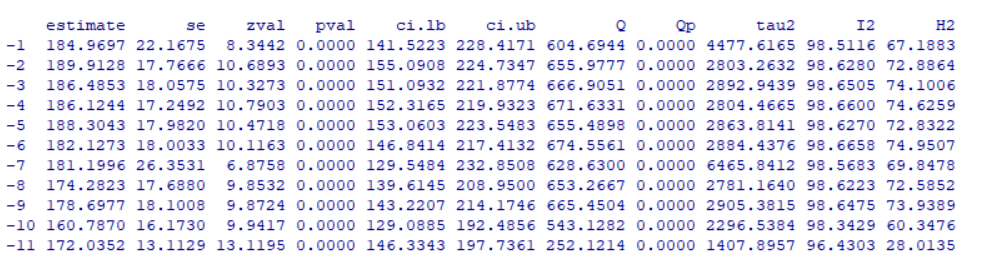


Duration of motor block


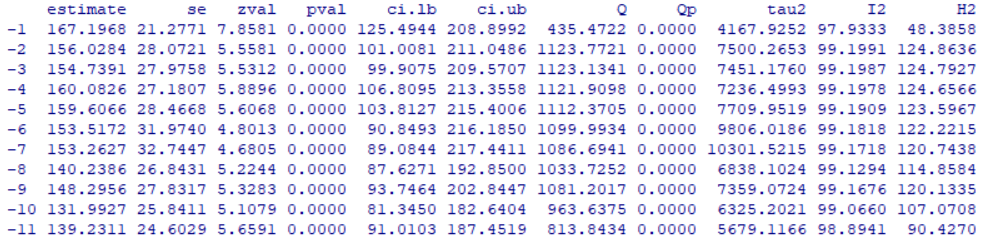


Onset of sensory block


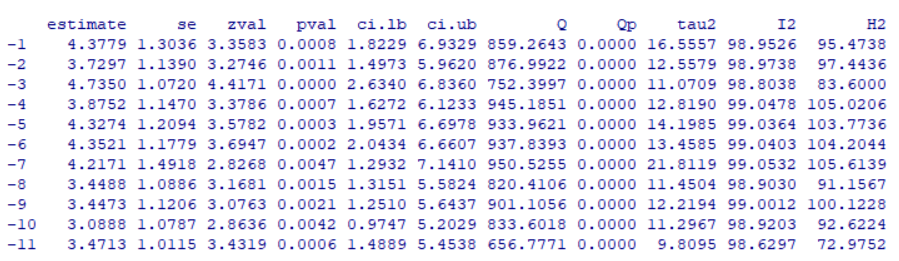


Onset of motor block


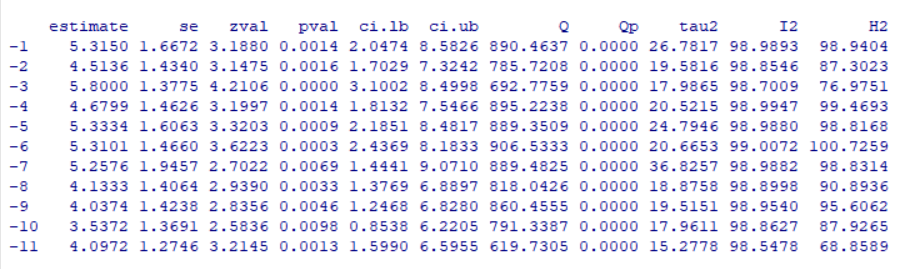


Total analgesic in 24h


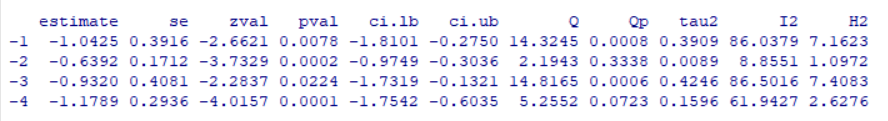

Supplement: Supplementary file 1 [file mmc1.docx]
